# Temporal and partial inhibition of GLI1 in neural stem cells (NSCs) results in the early maturation of NSC derived oligodendrocytes in vitro

**DOI:** 10.1186/s13287-019-1374-y

**Published:** 2019-08-27

**Authors:** Poommaree Namchaiw, Han Wen, Florian Mayrhofer, Olga Chechneva, Sangita Biswas, Wenbin Deng

**Affiliations:** 1 Department of Biochemistry and Molecular Medicine, University of California, Davis, Sacramento, CA USA; 2 School of Pharmaceutical Sciences, SunYat Sen University, Shenzhen, China; 3 Institute of Pediatric Regenerative Medicine, Shriners Hospital for Children, Sacramento, CA USA; 4 Institute of Medicinal Plant Development, Chinese Academy of Medical Sciences and Peking Union Medical College, Beijing, China

**Keywords:** Oligodendrocyte, GANT61, Myelination, NSC, Sonic Hedgehog, SHH, OPC, RNA-Seq, Differentiation, GLI1, hiPSC, hESC, Small molecule

## Abstract

**Background:**

Oligodendrocytes are a type of glial cells that synthesize the myelin sheath around the axons and are critical for the nerve conduction in the CNS. Oligodendrocyte death and defects are the leading causes of several myelin disorders such as multiple sclerosis, progressive multifocal leukoencephalopathy, periventricular leukomalacia, and several leukodystrophies. Temporal activation of the Sonic Hedgehog (SHH) pathway is critical for the generation of oligodendrocyte progenitors, and their differentiation and maturation in the brain and spinal cord during embryonic development in mammals.

**Methods:**

Our protocol utilized adherent cultures of human induced pluripotent stem cells (iPSC) and human embryonic stem cells (hESCs) with a green fluorescent protein (GFP) reporter knocked into one allele of the OLIG2 gene locus, dual SMAD inhibition, and transient partial inhibition of glioma-associated oncogene 1 (GLI1) by the small molecule GANT61 during the formation of the SOX2/PAX6-positive neural stem cells (NSCs). The SHH pathway was later restimulated by a Smoothened agonist purmorphamine to induce the generation of OLIG2 glial precursors. One hundred ninety-two individual oligodendrocyte precursor cells (OPCs) from GANT61 and control group were analyzed by single-cell RNA sequencing (RNA-Seq).

**Results:**

We demonstrate here that transient and partial inhibition of the SHH pathway transcription factor GLI1 in NSCs by a small molecule inhibitor GANT61 was found to generate OPCs that were more migratory and could differentiate earlier toward myelin-producing oligodendrocytes. Single-cell transcriptomic analysis (RNA-Seq) showed that GANT61-NSC-derived oligodendrocyte precursor cells (OPCs) had differential activation of some of the genes in the cytoskeleton rearrangement pathways that are involved in OPC motility and induction of maturation. At the protein level, this was also associated with higher levels of myelin-specific genes in the GANT61 group compared to controls. GANT61-NSC-derived OPCs were functional and could generate compact myelin in vitro and in vivo after transplantation in myelin-deficient shiverer mice.

**Conclusions:**

This is a small molecule-based in vitro protocol that leads to the faster generation of functional oligodendrocytes. The development of protocols that lead to efficient and faster differentiation of oligodendrocytes from progenitors provides important advances toward the development of autologous neural stem cell-based therapies using human iPSCs.

**Electronic supplementary material:**

The online version of this article (10.1186/s13287-019-1374-y) contains supplementary material, which is available to authorized users.

## Background

Oligodendrocytes (OLs) are a type of glial cells that are essential for the generation of the myelin sheath around the axonal processes in the mammalian central nervous system [1, 2]. Myelin is critically important for nerve conduction, axonal integrity and survival, and pathological changes in the oligodendrocyte metabolism can result in neurodegeneration. Before maturing into myelinating oligodendrocytes, oligodendrocyte precursor cells (OPCs) pass through multiple developmental stages, which can be characterized by their specific morphologies and expression of various transcription factors and cellular markers [1]. Activation of the Sonic Hedgehog (SHH) pathway is critical for the generation of oligodendrocytes. During embryonic development, SHH is secreted from the neural floor plate and forms a concentration gradient along the ventral notochord resulting in the switching of the ventral neural progenitor toward an oligodendroglial progenitor fate [3]. The activation of SHH signaling results in fate switching of neural stem cells (NSCs) to neural/glial antigen 2 (NG2)+/OLIG2+ motor neuron and OPCs [4–6]. Although the fundamental pathways of oligodendrocyte lineage commitment are highly conserved between human and mouse, still they do not fully recapitulate the timeline of human brain development. For example, during human embryonic development, gliogenesis begins at the second trimester and extends into adulthood [7, 8] while in rodent, it begins postnatally [8, 9]. Human induced pluripotent stem cell (iPSC) technology, on the other hand, allows us to complement our knowledge on human oligodendroglial development [10, 11]. Specifically, NSCs generated from human induced pluripotent stem cells (hiPSCs) and human embryonic stem cells (hESCs) have provided valuable mechanistic insights into the critical genes, transcription factors, morphogens, and regulatory elements that are activated during the generation of oligodendrocytes and subsequently their differentiation and myelination. iPSC-derived NSCs can be patterned in vitro toward a ventral spinal cord glial progenitor fate and driven to differentiate toward myelinating oligodendrocytes mainly through the activation of the SHH signaling pathways [12–14]. Specifically, the stimulation of SHH signaling activates its downstream effectors smoothened (SMO) and the glioma-associated oncogene homolog (GLI) family of zinc finger transcription factors that target several genes critical for oligodendrocyte specification and development [4, 15–19]. Previously, continuous stimulation of the SHH signaling pathway through either the Patch receptors or Smoothened receptors was used in most oligodendroglial differentiation protocols including our laboratory [12–14]. Other protocols have used the forced expression of transcription factors or microRNA expression to generate robust OPCs that can mature faster. For example, Ehrlich et al. [20] reported that the induction of the transcription factors SRY-box 10 (SOX10), oligodendrocyte transcription factor 2 (OLIG2), and NKX6.2 in iPSC-derived neural progenitors accelerates oligodendroglial differentiation significantly resulting in up to 70% of oligodendrocyte marker O4-positive (O4+) oligodendrocytes within 28 days. These oligodendrocytes can myelinate the CNS during development and after demyelination. Garcia-Leon et al. [21] demonstrated that overexpression of SOX10 was sufficient to generate O4+ and myelin basic protein (MBP)-positive (MBP+) OLs from human iPSCs in only 3 weeks, including from patients with amyotrophic lateral sclerosis and multiple sclerosis. The SOX10-induced O4+ population resembled primary human oligodendrocytes at the transcriptome level and could myelinate neurons in vivo. Nazari et. al. [22] reported that hiPSCs treated with microRNA (mRNA)-338 were differentiated into OPCs after treating for 16 days with basic fibroblast growth factor (bFGF), epidermal growth factor (EGF), and platelet-derived growth factor (PDGF)-AA. These protocols have an important use in many of the applications such as in vitro screening for drugs that promote OL maturation and myelination and disease modeling. Identifying the critical pathways that promote OPC generation and oligodendrocyte differentiation, and identifying small molecules that modulate that pathway, may provide an alternative protocol that carries a translation potential as well.

Interestingly, a fate-mapping study of adult NSCs in a cuprizone-induced demyelination mouse model revealed that there is a downregulation of the SHH pathway downstream of GLI1 in the NSCs residing in the CNS prior to their differentiation and mobilization to the demyelination site. Notably, these GLI-expressing and SHH-sensitive NSCs were the only subpopulation of NSCs within the subventricular zone (SVZ) that migrated and remyelinated the lesion [23]. They also observed that myelination started earlier during development in mice that had partial genetic ablation of GLI1 in NSCs [23]. This suggested that the temporal partial inhibition/activation of the SHH regulatory pathway (rather than the continuous activation) is required for oligodendrocyte development and maturation from NSCs in the adult mouse brain. In this study, we demonstrate that a transient and selective partial inhibition of GLI1 by the small molecule GLI1 inhibitor GANT61 in early stages of NSC formation followed by SHH pathway activation by the SMO agonist purmorphamine generated OLIG2-expressing OPCs that are more migratory and differentiated earlier than control OPCs. Partial inhibition of GLI1 by GANT61 during NSC induction from human iPSCs (referred to as GANT61 NSC-derived OPCs) results in greater upregulation of OPC and myelin-associated genes compared to controls during OPC differentiation. Single-cell RNA sequencing showed that the cytoskeletal reorganization pathways involved in morphological changes during oligodendrocyte differentiation and maturation pathways were activated earlier in GANT61 NSC-derived OPCs compared to control. GANT61-derived OLs were functional and could myelinate rat dorsal root ganglion (DRG) neurons in vitro and shiverer mouse corpus callosum.

## Materials and methods

### OLIG2-hiPSC and OLIG2-hESC cultures

We utilized two OLIG2-GFP knockin human pluripotent stem cell reporter lines (OLIG2-hESC and OLIG2-hiPSC) to follow the OLIG2+ precursors by live fluorescent imaging. The OLIG2-hESCs and OLIG2-hiPSCs have a GFP reporter inserted into one allele of the OLIG2 gene locus as previously described by our lab [24], which allow us to isolate OLIG2+ cells by the expression of GFP.

All experimental procedures conducted on hiPSCs and hESCs were approved by the University of California at Davis Stem Cell Research Oversight Committee. hiPSCs and hESCs were maintained in feeder-free and serum-free culture conditions on human pluripotent stem cell-qualified Matrigel matrix-coated (Corning, USA, growth factor-reduced type) 6-well plates using the mTeSR™1 medium (STEMCELL Technologies, USA) in 37 °C incubator with 5% CO_2_.

### Neural induction

**Fig. 1:**
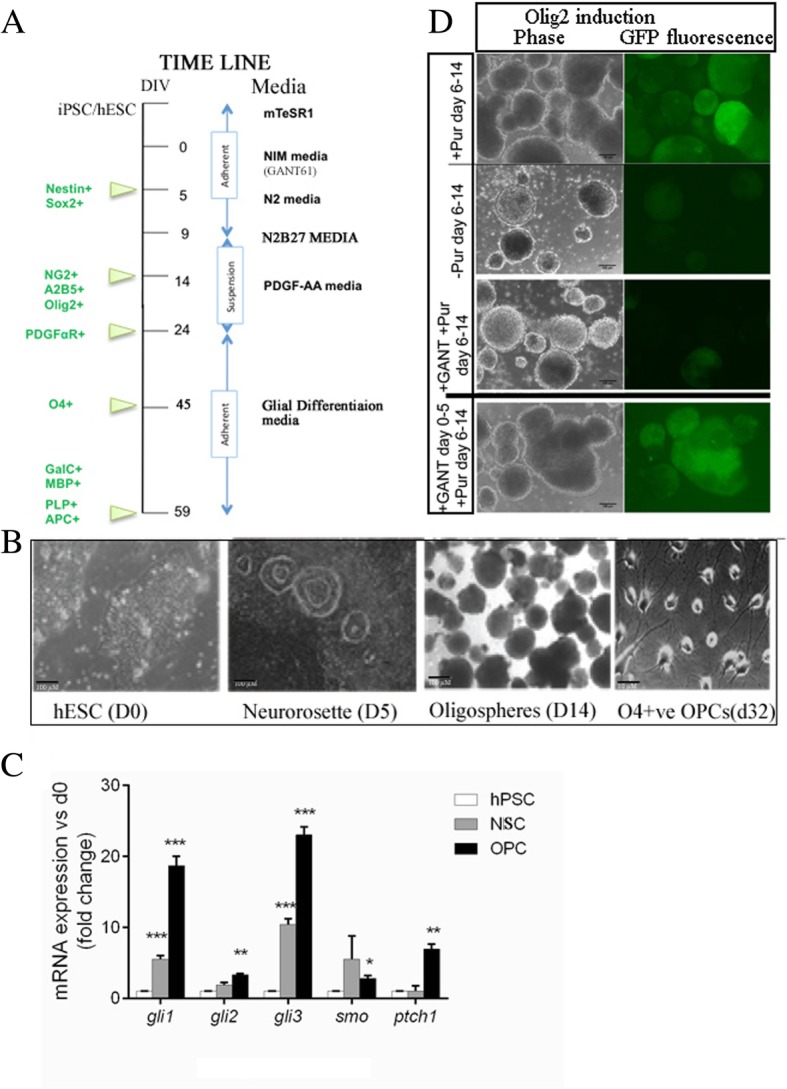
**a** The experimental timeline of oligodendrocyte differentiation from hiPSC colonies. **b** Phase bright images of iPSC colonies, neurorossette formation on day 5, oligospheres in suspension cultures, and O4+ Pre-OLs. **c** Expression pattern of SHH pathway genes in undifferentiated hiPSC, hiPSC-derived NSCs, and OLIG2-OPCs (three independent RT-PCR assays, *n* = 3, *t* test). **d** The generation OLIG2+/GFP+ glial progenitors (on day 14) following purmorphamine treatment in NSCs from day 6 to 14 (top row) and the absence of OLIG2/GFP induction without purmorphamine (second row); GANT61 treatment in NSC concomitant with purmorphamine (from day 6 to 14) did not give rise to OLIG2/GFP glial progenitors (third row), and GANT61 treatment from day 0 to 5 followed by purmorphamine (days 6–14) gave rise to OLIG2+/GFP+ glial progenitors (fourth row). The images in **d** is a representation of three independent experiments. Scale bar, 100 μm. Error bars indicate SEM. ***P < 0.001; **P < 0.01; *P < 0.05 vs. day 0

A schematic diagram of the timeline of OLIG2-NSC and OLIG2-OPC generation and differentiation is shown in Fig. 1a. First, in the neural induction phase, we generated NESTIN^+^/SOX2^+^ NSCs (generated from neural rosette-like structures in adherent cultures) by exposing adherent cultures of hiPSCs from day 0 to 5 to the neural induction media (NIM) containing all-trans retinoic acid (RA, 0.1 μM), and dual SMAD signaling inhibitors SB431542 (10 μM) and LDN193189 (250 nM) [12, 25]. Beginning on day 6, cells were cultured in N2 media containing all-trans RA (0.1 μM), and the SMO agonist purmorphamine (1.0 μM) added fresh daily to the medium to pattern these NSCs to mimic the ventral spinal cord OLIG2^+^/pre-motor neuron domain. The cells were cultured in N2B27 media containing RA (0.1 μM) and purmorphamine (1.0 μM) from day 9. The induction of OLIG2 expression was tracked visually by the appearance of GFP expression under a fluorescence microscope (Fig. 1d, top panel). On day 14, the OLIG2-GFP-positive cells were detected and were further enriched and matured by expansion as oligospheres in ultralow attachment plates in PDGF-AA medium. Non-GFP-expressing cells were unable to form spheres and were excluded by centrifugation. OPC maturation was induced by replacing the PDGF-AA medium by the glial differentiation medium (GDM) under adherent culture in poly-d-lysine/growth factor-reduced Matrigel-coated dishes or coverslips.

### Pharmacological inhibition of GLI1

We have used GANT61 for partial inhibition of Gli1 transcriptional activity based on our own pilot study and on published data. GLI1 is a specific target for the small molecule inhibitor, GANT61, which binds directly to the GLI1 protein and not to the DNA or to other zinc finger transcription factors. GANT61 is a hexahydropyrimidine derivative, namely 2-[[3-[[2-(dimethylamino)phenyl]methyl]-2-pyridin-4-yl-1,3-diazinan-1-yl]methyl]-*N*,*N*-dimethylaniline (molecular formula is C27H35N5 and molecular weight is 429.60). It is soluble in DMSO and ethanol. GANT61 can efficiently block GLI1 transcription. In our case, we observed a modest (~ 30%) cytotoxicity between 5 and 10 μM, and the EC50 was 1.75 (Additional file 1: Figure S1.A). In our pilot study, we found that 5 μM and 10 μM GANT61 reduced the mRNA expression of GLI1 by 30% and 60%, respectively, in NSCs (Additional file 1: Figure S1B). Also, 5-μM GANT61 could prevent the induction of SHH pathway-dependent olig2 expression in NSCs (as shown earlier in Fig. 1d, third panel). Thus, the concentration of 5 μM GANT61 was selected based on the viability curve and the reduction of GLI1 mRNA expression (Additional file 1: Figure S1.A and B). In one set, GANT61 (5 μM), a selective SHH-GLI1 inhibitor was added during the neural induction phase in the NIM medium from day 0 to 5 along with the dual SMAD inhibitors and RA. On day 6, GANT61 was withdrawn and the SHH pathway was restimulated by adding the SMO agonist purmorphamine (1 μM along with RA (0.1 μM)) for the generation of OLIG2^+^ glial progenitors (from day 6 to 14). In another set, GANT61 (5 μM) was applied during the glial induction phase (day 6–14). Specifically, GANT61 was added along with purmorphamine on days 6–14 of the differentiation timeline. OPCs from both control and GANT6 groups were expanded as oligospheres in PDGF-AA medium.

### Immunofluorescence staining

The cells were cultured on poly-d-lysine/Matrigel-coated (growth factor-reduced) coverslips. The immunofluorescence staining of hPSC-derived NSCs, OPCs, and oligodendrocytes was performed using the following primary antibodies: anti-NESTIN (Santa Cruz Biotechnology, USA), anti-sex determining region Y-box 2 (SOX2) (Millipore, USA), anti-NG2 (Santa Cruz Biotechnology, USA), anti-GFP (Millipore, USA), anti-PDGFR-α (Santa Cruz Biotechnology, USA), anti-galactosylceramidase (GALC) (Santa Cruz Biotechnology, USA), anti-O4 (R&D Systems, USA), anti-MBP (R&D Systems, USA), anti-myelin oligodendrocyte glycoprotein (MOG) (Santa Cruz Biotechnology, USA), anti-proteolipid peptide (PLP1) (Santa Cruz Biotechnology, USA), and anti-Glial fibrillary acidic protein (GFAP) (Santa Cruz Biotechnology, USA) as per manufacturer’s recommendations. Alexa Fluor 488, 545, 595, or 647 conjugated secondary antibodies (Thermo Fisher Scientific, USA, 1:1000) were used. In brief, coverslips were washed with Dulbecco’s phosphate-buffered saline (DPBS) and fixed with 4% paraformaldehyde (PFA) for 15 min at room temperature. The cells were permeated by 0.25% Triton X-100 for 10 min at room temperature and blocked with 1% bovine serum albumin (BSA) in DPBS. The primary antibodies were stained overnight at 4 °C and washed three times prior to secondary antibody staining for 30 min at room temperature. The coverslips were mounted with Fluoromount-G containing the nuclear stain DAPI (Southern Biotech, USA). The images were taken by a Nikon C1 confocal microscope.

### Flow cytometry

Cells were stained with different oligodendrocyte lineage markers according to standard published protocols for flow cytometry. In brief, GFP^+^/OLIG2^+^ OPCs were enzymatically digested by Accutase enzyme mix (STEMCELL Technologies, USA) to obtain single cell suspension. Cells were then pelleted by centrifugation at 500*g* for 5 min at 4 °C and then resuspended at a concentration of 1–5 × 10^6^ cells/mL in ice-cold 10% BSA in DPBS solution. Cells were then strained through a 40-μm cell strainer prior to flow cytometry. Analysis of GFP expression and various oligodendrocytes markers was performed on the Attune NxT Acoustic Focusing Cytometer (Thermo Fisher Scientific, USA), and data were analyzed with the AttuneTM NxT software. Background fluorescence was excluded using unstained vehicle control OPCs. For each sample, more than 10,000 events were recorded and were used to evaluate the percentage of GFP-expressing cells and different OPC and oligodendrocyte population.

### Viability assay

Double live/dead cell staining buffer containing calcein-AM and propidium iodide (Dojindo Molecular Technologies, USA) was added to the cell. A fluorescence plate reader detector (Perkin Elmer, USA) for 96-well plates was used to measure the live/dead cells and calculate the relative cell viability. In a separate set of experiments, propidium iodide (PI) dye inclusion method was used to identify dead cells measured by the Attune NxT Acoustic Focusing Cytometer (Thermo Fisher Scientific, USA). Live/dead cell double staining can be utilized for simultaneous fluorescence staining of viable and dead cells. Calcein-AM is a highly lipophilic and cell membrane-permeable dye. Though calcein-AM itself is not a fluorescent molecule, the calcein generated from calcein-AM by esterase in a viable cell emits a strong green fluorescence (*λ*ex 490 nm, *λ*em 515 nm). Therefore, calcein-AM only stains viable cells. Alternatively, the nuclei staining dye propidium iodine cannot pass through a viable cell membrane. It reaches the nucleus by passing through disordered areas of the dead cell membrane and intercalates with the DNA double helix of the cell to emit red fluorescence (*λ*ex 535 nm, *λ*em 617 nm). Since both calcein and PI-DNA can be excited with 490-nm light, simultaneous monitoring of viable and dead cells is possible with a fluorescence microscope.

### Cell proliferation assay

Cell proliferation was assayed by the SYTOX® Blue dead cell stain kit (Invitrogen, USA). SYTOX® Blue is a high-affinity nucleic acid stain that penetrates cells with compromised plasma membranes but will not cross intact cell membranes. After incubation with 1 μM SYTOX® Blue stain for 5 min at room temperature, protected from light, the nucleic acids of dead cells fluoresced bright blue when excited with 405-nm violet laser light. The cells were then analyzed without fixing with either a 440/40-nm or a 530/30-nm band-pass filter with the Attune NxT Acoustic Focusing Cytometer (Thermo Fisher Scientific, USA). In another set, the rate of proliferation was assessed after the incubation period following staining with CellTrace Violet (Thermo Scientific, USA) according to the manufacturer’s instructions.

### Cell migration

Oligospheres (expressing PDGFR-α at day 34 of differentiation) of similar sizes from both control and GANT61 groups were plated on poly-d-lysine/Matrigel-coated (growth factor reduced) glass coverslips in GDM medium. The spheres were allowed to attach to the coverslips for 2 h in few drops (~ 100 μL) of the medium in a petri dish. After 2 h, the coverslips were placed into individual wells of a 24-well plate with fresh 1 mL GDM. After 2, 24, and 48 h of plating, 15 oligospheres from each group were imaged, and the distances traveled by various cells were imaged under an inverted microscope and measured and analyzed by ImageJ.

### The real-time semiquantitative PCR (RT-PCR) analysis

Total RNA was extracted from the cells in cultures using the RNeasy Mini Kit (Qiagen, USA). cDNA was synthesized using the qScriptTM XLT cDNA SuperMix (Quanta Bio, USA), and qPCR was performed using the PerfeCTa® SYBR® Green SuperMix and Low ROX (Quantabio, USA) running on the MX30005p light cycler system (Stratagene, USA). Expressions of all the genes were normalized to the expression of the housekeeping gene GAPDH. The list of the primers (Invitrogen, USA) is included in Additional file 6: Table S1.

### Dorsal root ganglion neuron co-culture with GANT61 derived OPCs

Rat DRGs (Lonza Inc., USA) were plated on collagen type I-coated coverslips and allowed to mature for 2 weeks in primary neuronal growth medium (Lonza Inc., USA). Rare contaminations of rat OPCs were eliminated by continuous exposure to medium containing 5-fluorodeoxyuridine 0.1 μM and uridine 0.1 μM. GANT61-NSCs were differentiated to PDGFR-α-expressing OPCs. OPCs were then plated on top of the DRG neurons and cultured in GDM medium. After 3 weeks of co-culture, the cells were fixed and processed for electron microscopy (EM) imaging. All experiments were performed in accordance with relevant guidelines and regulations set by the University of California, Davis.

### Transplantation of GANT61-derived OLIG2/GFP-OPCs in shiverer mouse brain

Shiverer mice (C3Fe.SWV-Mbpshi/J, The Jackson Laboratory, USA) lack MBP and compact myelin in the CNS. Therefore, these mice serve as a good model to test the functional capacity of transplanted OPCs toward forming MBP-positive compact myelin sheath around axons. Shiverer mice 8–10 weeks old (*N* = 3; 2 males, 1 female) were injected with 50,000 iPSC-derived GANT61-OLIG2^+^/GFP^+^/PDGFR-α^+^ OPCs. Mice were deeply anesthetized by a cocktail of ketamine (70 mg/kg) and xylazine (10 mg/kg). Then, 1 μL of cell suspension was injected aseptically (at the rate of 0.3 μL/min) into the corpus callosum using a Hamilton syringe at the following stereotaxic coordinates: anteroposterior 0.2, mediolateral 1.1 with Bregma as a reference, and dorsoventral 2 mm from the skull surface. Mice were administered cyclosporin 10 mg/kg daily intraperitoneally for immune suppression, beginning 2 days before cell transplantation until sacrifice. Postoperative care was given according to the Institutional Animal Care and Use Committee (IACUC) protocols approved by the University of California, Davis. Mice were sacrificed after 4 or 6 weeks, and the corpus callosum sections (2–3 mm) containing the injection site was microdissected and processed for imaging under an electron microscope or for confocal imaging.

### The single-cell RNA sequencing (RNA-Seq)

Oligospheres at day 39 (OLIG2/GFP+ spheres were actually collected at day 24 of differentiation and expanded in PDGF-AA media for 2 weeks in suspension) were dissociated into single cells using the cell dissociation enzyme Accutase (STEMCELL Technologies, USA). Briefly, Accutase was added to the culture wells containing the spheres and incubated at room temperature for 5–10 min. Next, the culture dishes were placed in a cell culture incubator at 37 °C for 5 min for the inactivation of Accutase enzyme. Spheres were then gently triturated with a glass pipette to dissociate the spheres into single cells. Then, they were passed through a 40-μm cell strainer to obtain single cell suspension. The single-cell RNA library was prepared by sorting the single cells onto a 96-well plate prefilled with BD™ Precise Library Prep Kit Buffer. Fluorescence-activated cell sorting (FACS) was done by the UC Davis Flow Cytometry Core Facility. Ninety-six individual OPCs from the control group and 96 GANT61 OLIG2/GFP-OPCs were sequenced. The sequencing was carried out on the NextSeq Illumina platform using a 75-bp paired-end, and a speed of 350,000 reads per cells, by UC Davis Genome Center core services. The sequence reads were analyzed on the Seven Bridges Genomics Platform using a WTA Precise computation pipeline and were aligned to the GRCh37/hg19 human reference genome. The sequence reads were normalized with the Bioconductor R packages zero-inflated negative binomial model (ZinB) [26] prior to determining the differential gene expressions (DEGs). One thousand highly variable genes were recruited for DEG analysis by the Empirical Analysis of Digital Gene Expression Data in R (EdgeR) software [27]. Subsequent Gene Ontology (GO) of the DEGs was performed using the Database for Annotation, Visualization and Integrated Discovery bioinformatics resource [28].

### Cell culture media

The following are the cell culture media:

- mTeSR1: mTeSR1 basal medium was supplemented with 50× mTeSR1 supplement and 100× penicillin-streptomycin to get the final concentration of 1× each.
- Basal medium: DMEM/F12 supplemented with non-essential amino acids (NEAA) 1×, GlutaMAX 2 mM, 2-mercaptoethanol, and penicillin-streptomycin to get the final concentration of 1x each.
- Neural Induction Medium (days 0–5): basal medium supplemented SB431542 10 μM () LDN193189 (250 nM) and RA (0.1 μM).
- N2 medium: basal medium supplemented with N2 supplement (Gibco, USA, 100× stock) to get a final concentration of 1× N2, laminin 4 μg/mL (Roche, Switzerland), bFGF 10 ng/mL (Peprotech, USA), and Noggin 20 ng/mL (Peprotech, USA). RA 0.1 μM (Sigma-Aldrich, USA) and purmorphamine 1 μM (Cayman, USA) were added fresh during medium change.
- N2B27 medium (days 10–14): basal medium containing 1× of N2 (Gibco, USA, 100× stock) and 1× of B27 (Gibco, USA, 50× stock) supplements. RA was removed in this step. One micromolar purmorphamine was added fresh during media change.
- PDGF-AA medium: basal medium containing 1× N2 (Gibco, USA, 100× stock), 1× B27 (Gibco, USA, 50× stock) supplements, biotin 100 ng/mL (Sigma-Aldrich, USA), PDGF-AA 10 ng/mL (Peprotech, USA), IGF-1 10 ng/mL (Peprotech, USA), T3 60 ng/mL (Sigma-Aldrich, USA), NT3 10 ng/mL (Peprotech, USA), HGF 5 ng/mL (Peprotech, USA), and cAMP 1 μM (Sigma-Aldrich, USA).
- Glial differentiation medium (GDM): basal medium containing 1× N2, 1× B27 supplements, biotin 100 ng/mL (Sigma-Aldrich, USA), T3 60 ng/mL (Sigma-Aldrich, USA), ascorbic acid 20 μg/mL (Sigma-Aldrich, USA), NT3 10 ng/mL (Peprotech, USA), HEPES 10 mM (Sigma-Aldrich, USA), cAMP 1 μM (Sigma-Aldrich, USA), Y-27632 10 μM (Cayman, USA), and heregulin 10 ng/mL (Peprotech, USA).

### Statistical analysis

Statistical analyses are performed using the independent *t* test for two-sample comparison or one-way analysis of variance (ANOVA) with post hoc Bonferroni test for three-sample comparison (Prism6, version 6.01). The experiments were done in triplicate unless mentioned otherwise. Values are expressed in each graph as the mean ± SEM. The differences between mean values for different treatments are considered to be significant at *P* < 0.05 (presented as “*”), at *P* < 0.01 (presented as “**”), and at *P* < 0.001 (presented as “***”).

## Results

### SHH signaling pathway genes are differentially expressed in the hiPSCs, hiPSC-derived NSCs, and hiPSC-derived OPCs

We evaluated the mRNA expression of key SHH pathway genes in hESCs, NSCs, and OPCs generated according to the timeline (Fig. 1a). Human iPSC cell line and the hESC line were grown on Matrigel-coated plates in E8 media and then differentiated into NSCs and olig2-expressing oligospheres and finally OPCs (Fig. 1b). Specifically, we measured the mRNA expression levels of the SHH signaling receptors Smoothened (*SMO*) and Patched1 (*PTCH1*), and their downstream effector’s transcription factors (TFs) *GLI1*, *GLI 2*, and *GLI3* (Fig. 1c).

We detected low levels of mRNA *GLI1*, *GLI 2*, and *GLI3* in undifferentiated hiPSCs (*n* = 3) as expected since the GLI TFs are thought to play a minimal role in the maintenance and self-renewal of hiPSCs and hESCs (Fig. 1c). Next, we looked at the expression levels of the mRNA for *GLI1*, *GLI 2*, *GLI3*, Smoothened (*SMO*) and Patched1 (*Ptch1*) in hiPSC-derived NSCs that were generated after hiPSCs were exposed to RA and dual SMAD inhibitors in NIM media at day 5 (Fig. 1c). We found that in the SOX2^+^ and NESTIN^+^ NSCs, *GLI1* and *GLI3* mRNAs were upregulated by 5.5- and 9-folds, respectively, compared to undifferentiated hiPSCs (*P* < 0.001 for both *GLI1* and *GLI3*) (Fig. 1c). The expression of *GLI2* was not significantly different between undifferentiated hiPSCs and derived NSCs (Fig. 1c). A similar expression pattern was also observed in the OLIG2-hESC-derived NSCs (shown in Additional file 1: Figure S1.C). Thus, we confirmed that SHH signaling pathway genes were highly transcribed during the differentiation of hPSC toward the NSCS. Other studies have also shown that the SHH is a critical regulator of NSC formation and later their specification toward OPC fate during mammalian brain development [3, 6, 19, 29–31].

Next, we patterned the hiPSC-derived NSCs with RA and the Smoothened agonist purmorphamine to mimic the ventral spinal cord NSCs which generate OLIG2^+^ progenitors (green cells) expanded as oligospheres (Fig. 1b). OLIG2+ progenitors differentiate into motor neurons and later OPCs in vitro and in vivo. At day 14 of the differentiation timeline, GLI1, GLI3, and Ptch1 mRNA levels in early OPCs were further upregulated compared to NSCs measured at day 5 (*P* < 0.05, *P* < 0.01, and *P* < 0.001, respectively) (Fig. 1c). These results indicated that the SHH pathway genes were differentially transcribed during the neural and glial induction phases.

Partial inhibition of GLI1 by GANT61 in NSCs during the glial induction phase (even in the presence of the Smoothened agonist) inhibited the generation of OLIG2+ glial progenitors in vitro

In an effort to investigate the effect of partial inhibition of the SHH pathway on OL development and maturation, we first sought to explore the effects of partial inhibition of GLI1 during the glial induction phase. Typically, the stimulation of the SHH pathway in the NSCs (in our case with the Smoothened agonist purmorphamine during days 6–14) is critical for glial induction in vitro (seen by the generation of OLIG2/GFP-positive glial progenitors) (Fig. 1d, top panel), since OLIG2/GFP progenitors were not generated in vitro in the absence of purmorphamine (Fig. 1d, second row). Therefore, we treated the NSCs with GANT61 during the glial progenitor induction phase (days 6–14 of the differentiation timeline). We found that 5 μM GANT61 prevented the generation of OLIG2^+^/GFP^+^ OPCs (Fig. 1d, third row), to a level that is seen in the complete absence of purmorphamine. Thus, we concluded that partial inhibition of GLI1 signaling pathway during glial induction inhibited the generation of glial progenitors.

### Partial inhibition of GLI1 by GANT61 during neural induction phase results in greater upregulation of OPC and oligodendrocyte genes compared to controls OPC

**Fig. 2:**
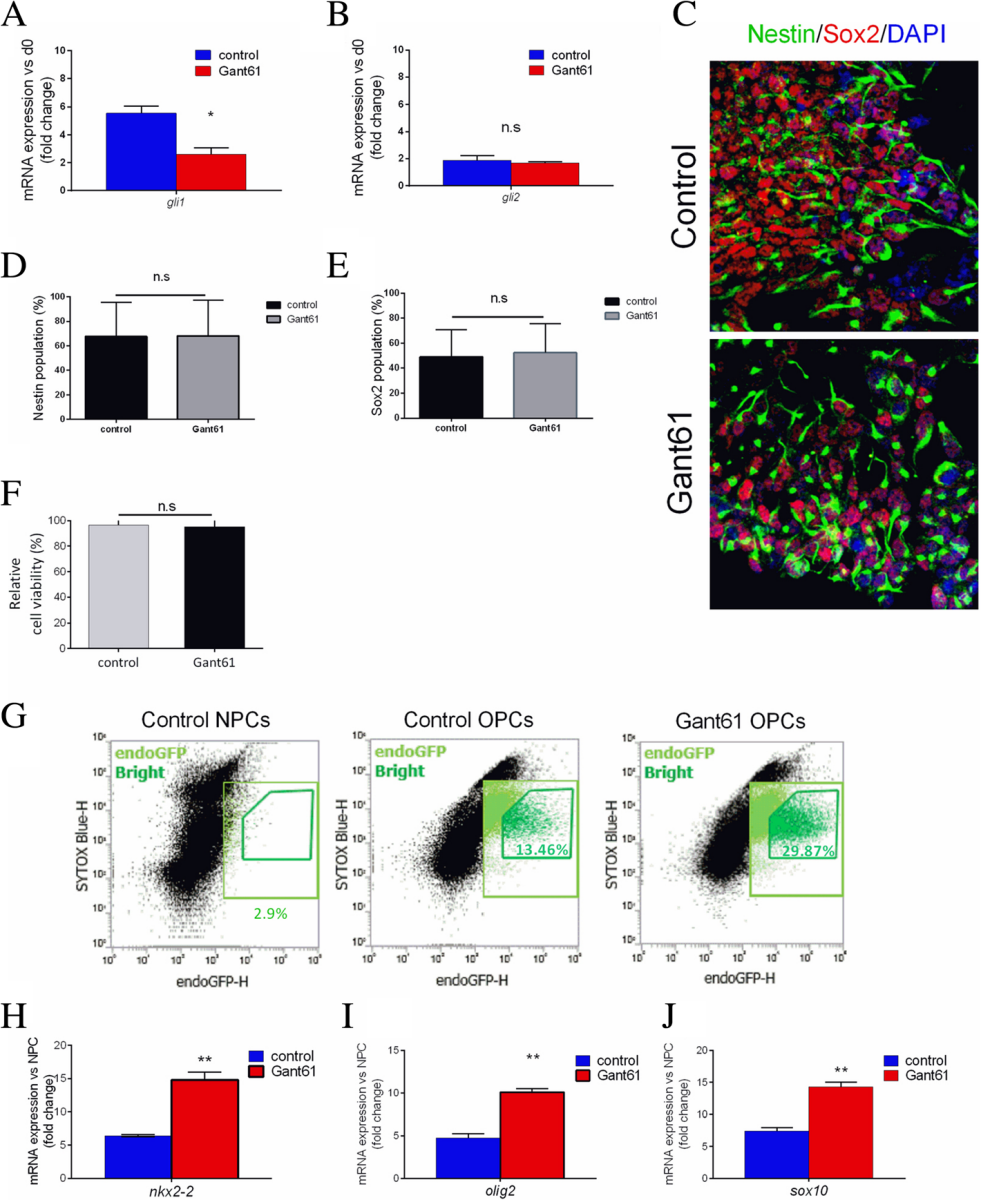
The relative mRNA expression at day 5 of **a** *GLI1* and **b** *GLI2* vs. undifferentiated hiPSCs in the absence and presence of GANT61 (*n* = 3, *t* test). **c** Representative immunocytochemistry images of NESTIN+/SOX2+ in control and GANT61-NSCs (day 5). Quantification of **d** NESTIN and **e** SOX2+ cells in control-NSCs and GANT61-NSCs (at day 5 of differentiation, *n* = 5 independent experiments). **f** Analysis of cell survival following GANT61 treatment from day 0 to 5 (assayed at day 5, *n* = 5). **g** A representative flow cytometry data of GFP/OLIG2 expression in NSC-derived OPCs (day 14, *n* = 3 independent experiments). **h–j** The gene expression of the transcription factors, NKX2.2 OLIG2 and SOX10, in GANT61-OPCs compared to control-OPCs (assayed at day 14, *n* = 3, *t* test.). Data are shown as fold change relative to the expression in NSC (*n* = 3, *t* test). Error bars indicate SEM. ***P < 0.001; **P < 0.01; *P < 0.05

Next, we partially inhibited GLI1 early during the neural induction phase (days 1–5). hiPSCs were treated with 5 μM GANT61 or dimethyl sulfoxide (DMSO) vehicle control together with all-trans RA and dual SMAD inhibitors for neural induction. We observed that *GLI1* mRNA levels were significantly reduced in the NSCs that were generated at day 5 (*P* < 0.001 compared to control NSCs) but *GLI2* levels were not different compared to control NSCs (Fig. 2a, b, respectively).

We then sought to characterize and differentiate these NSCs that were generated in the presence of transient partial *GLI1* inhibition (refer to as GANT61-NSCs). We found that both GANT61-NSCs and control-NSCs (DMSO vehicle control) differentiated into NESTIN^+^/SOX2^+^ NSCs with similar efficiency (Fig. 2c–e). The populations of Nestin^+^ and Sox2+ NSCs were similar between control-NSCs and GANT61-NSCs (Fig. 2d, e). GANT61-NSCs also showed no difference in cell viability and rate of proliferation compared with control NSCs (Fig. 2f, Additional file 3: Figure S3A). Similar results were seen with hESC-derived NSCs (Additional file 1: Figure S1.D and E).

We observed that the GANT61-NSCs were able to give rise to OLIG2^+^/GFP^+^ OPCs, which could be maintained and expanded as GFP+ oligospheres (green spheres) (Fig. 1c first and second row, also see SHH genes expression in Additional file 2: Figure S2.A-E). Similar results were seen in the Olig2-knockin H9 hESC line (Additional file 4: Figure S4.A). We used flow cytometry to count the percentage of OLIG2-GFP+ and NG2 co-expressing glial cells in control-NSCs and GANT61-NSCs (*n* = 3). The NG2^+^ OPC population is considered as the oligodendrocyte progenitors in the mammalian CNS [5]. We found that the population of NG2+/GFP (OLIG2)^+^ was not significantly different between both groups (see Additional file 2: Figure S2.F and G). However, we noticed a difference in the intensity of GFP expression (Fig. 2g). Since the GFP gene was knocked-in under the *OLIG2* locus in hiPSCs (and hESCs), the green fluorescence is a read-out of *OLIG2* promoter activity and *OLIG2* gene expression. In the NSC that were patterned toward OPC, two populations of GFP^+^ OPCs were identified in both GANT61-OPC and control-OPC (day 14), one with a lower level of GFP expression and the other with relatively brighter GFP expression. Whereas, control-NSCs (day 9) had a nominal fraction of cells (~ 3% of all events, *n* = 3) that expressed GFP^+^ (Fig. 2g, left panel). In control OLIG2-OPCs, 13.6 + 1.14% of all GFP+ cells were GFP bright while in GANT61-OPCs, 30.0 + 1.4% were GFP-bright cells (*P* < 0.001, *n* = 3, conducted three independent experiments, Fig. 2g) with no significant differences in the cell viability, but had slightly lower proliferation rate (Additional file 3: Figure S3.B). To test if the greater GFP-bright population reflects higher *OLIG2* mRNA expression, we assayed mRNAs of FACS-sorted GFP^+^ OPCs (day 14) by RT-PCR and found that GANT61 OLIG2-OPCs expressed higher levels of mRNAs for the key OPC transcription factor genes *OLIG2*, *SOX1*0, and *NKX2.2* compared to control OLIG2-OPCs (Fig. 2h–j). This indicated that early transient inhibition of GLI1 in NSCs leads to a greater upregulation of the critical oligodendrocyte developmental genes later in the presence of Smoothened agonist purmorphamine.

The higher levels of OPC-related gene expression and an increase in the GFP-bright subpopulation (higher reporter mRNA and protein expression indicating higher activation of OLIG2 gene) prompted us to hypothesize that the pattern and timeline of differentiation of OPCs from hiPSCs (or hESCs) is a program that may be already in place during the early phases of NSC generation, and maybe modulated by GLI1 early during NSC formation.

**Fig. 3:**
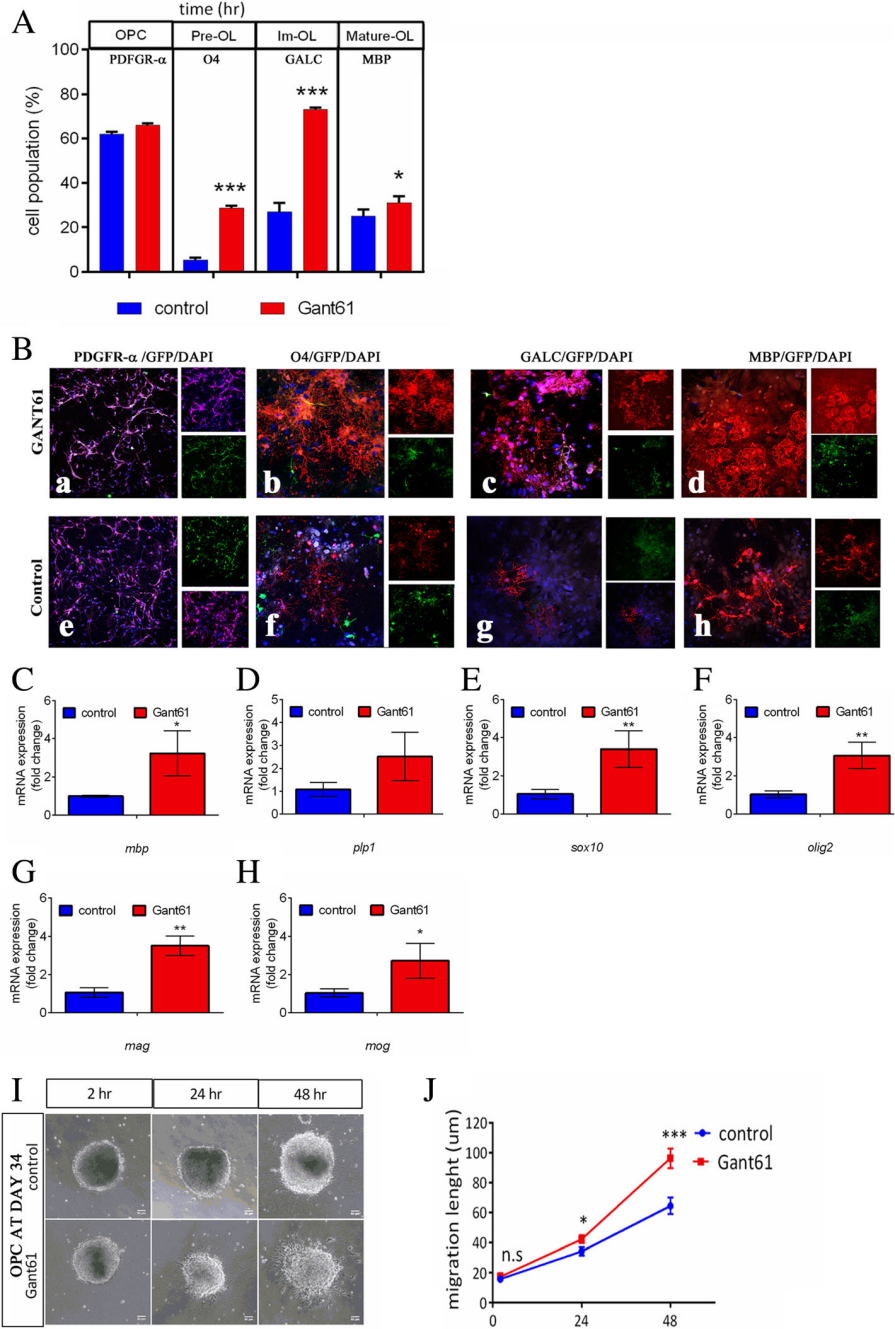
**a** Percentages of PDGFR-α+ OPCs (day 34), O4+ pre-oligodendrocytes (days 45–47), GALC+ immature oligodendrocytes (day 59), and MBP+ mature oligodendrocytes (day 59) (*n* = 3, three independent experiments). **b** The representative immunocytochemistry images of oligodendroglial protein expressions of PDGFR-α, O4, GALC, and MBP (three independent experiments were done). **c**–**h** The mRNA levels of MBP, PLP1, SOX10, OLIG2, *MAG*, and *MOG* at day 59 of oligodendrocyte differentiation timeline (*n* = 3). **i** Representative images of cells migrating out of spheroid at 2, 24, and 48 h after attachment to the culture plate (three independent experiments were done). **j** Quantification of cell migration distance (*n* = 3, ANOVA followed by *t* test). (Scale bar = 100 μm; *n* = 15, *t* test). Error bars indicate SEM. *P < 0.05; ***P < 0.001

Next, to study the effect of GANT61 on OL differentiation, we differentiated the control and GANT61 OLIG2-OPCs and followed the percentage of cells at different stages of maturation by the expression of the stage-specific immune markers by flow cytometry (Fig. 3a). Similar numbers of OLIG2-OPCs from control and GANT61 groups were initially plated on the poly-d-lysine/Matrigel-coated (growth factor-reduced) dish at 25% confluence and differentiated for up to 59 days. The percentage of PDGFR-α^+^ OPCs was similar in both groups at days 34–35 of the differentiation timeline. The late OPC marker O4 (referred to as Pre-OL) was detected as early as day 45 (31.5 + 1.5% in GANT61 compared to 6.5 + 1.1% in the control group (*P* < 0.001), *n* = 3). At day 59 of differentiation timeline, the fraction of cells expressing immature oligodendrocytes markers GALC and mature oligodendrocyte marker MBP in the GANT61 group was 74.2 + 0.5% and 31.3 + 0.5%, respectively, compared to control cells which were 31.2 + 0.7% and 25.4 + 0.6%, respectively (*P* < 0.001 for both markers, *n* = 3). The immunofluorescence staining of oligodendroglial markers was done at the same time frame (Fig. 3). We also repeated the study in the olig2-knocked-in H9 hESC line and found that the percentages of the PDGFR-α-expressing cells at day 34 in both GANT61 and control were 74.5 + 2.4% and 73.3 + 2.6%, respectively, while the percentage of GALC-expressing mature OL was significantly higher in the GANT61 group at day 59 (48.1 + 2.0 vs. 13.4 + 4.1 in control, *n* = 3, see Additional file 4: Figure S4.B and C).

At day 59 of differentiation, GANT61-derived oligodendrocytes showed upregulated levels of *SOX10* and *OLIG2* by 3.4-folds (*P* value = 0.005) and 2.6-folds (*P* value = 0.003), respectively, compared to the control-derived oligodendrocytes (RT-PCR assay, *n* = 3) (Fig. 3c, d). This was associated with a concurrent increase in myelin gene, i.e., myelin-associated glycoprotein (MAG) by 3.5-fold (*P* value = 0.005, *n* = 3), MOG by 2.7-folds (*P* value = 0.014, *n* = 3), and MBP by 3.2-fold (*P* value = 0.039, *n* = 3), (Fig. 3e–h). Together, these results indicate that around day 59, control OLIG2-OPCs show limited maturation whereas GANT61-derived OLIG2-OPCs were capable of maturing toward advanced stages by inducing the expression of critical transcription factors and myelin genes.

### Transient inhibition of *GLI1* during NSC formation later promotes the generation of OPCs that are more migratory than control OPCs

The migratory capacities of the PDGFR-α^+^ GANT61 OLIG2-OPCs and control OLIG2-OPCs were tested on poly-d-lysine/Matrigel-coated surfaces by measuring the distance traveled by the OPCs radiating out of oligospheres after attachment for 2, 24, and 48 h (Fig. 3i). The initial diameters of the plated oligospheres ranged from 200 to 300 μm and were similar between the two groups (*P* value = 0.15). The results show that after 48 h, the average distance of cell migration from GANT61 oligospheres (96.2 ± 6.5 μm) was significantly greater than vehicle control-oligospheres (64.3 ± 6.1 μm, *P* < 0.001)(Fig. 3j). Similar results were seen with H9 OLIG2-GFP knockin hESC-derived OPCs (GANT61 treatment from day 0 to 5, Additional file 4: Figure S4.D and E). In the OLIG2-GFP hESC line, the average distance of migration was 107 + 4.0 μM, while in the control group, the distance was 61.0 + 5.5 μM (*P* < 0.001). This result indicates that early transient inhibition of *GLI1* during NSC induction followed by reactivation of the SHH pathway by purmorphamine gave rise to the OPCs with relatively greater migratory capacity after glial differentiation.

### GANT61-derived oligodendrocytes showed upregulation of cytoskeletal reorganization pathway revealed by single-cell whole transcriptomic analysis

**Fig. 4:**
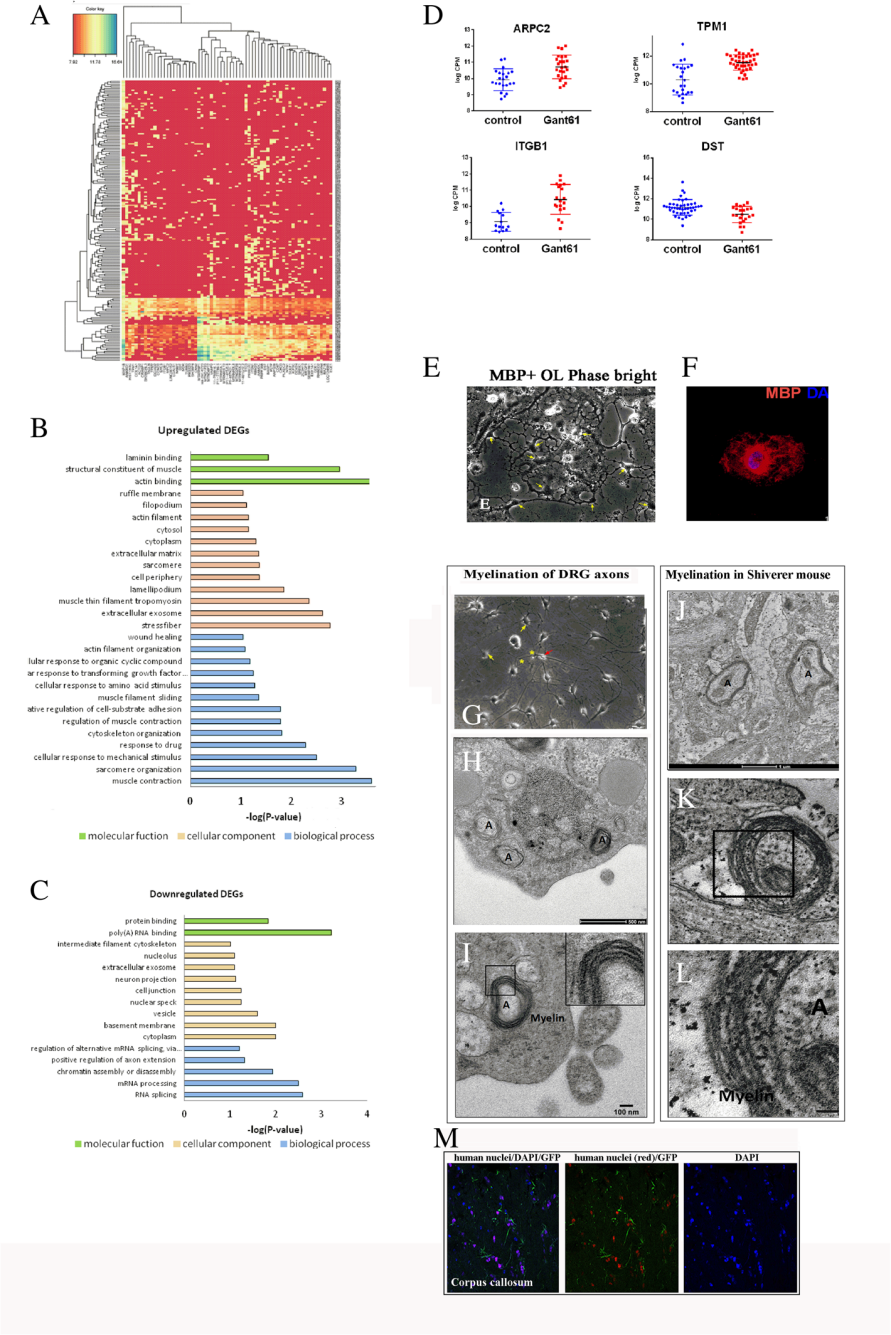
**a** Heat map of top differentially expressed genes (DEGs) expressed in hiPSC-derived oligodendrocytes (representative map from three independent experiments). Representative Gene Ontology (GO) of **b** upregulated genes and **c** downregulated genes in GANT61-OPCs compared to control OPCs (*n* = 192). **d** The expression level of significant DEGs of interest from the single-cell transcriptomic analysis in GANT61 and control OPCs (*n* = 192). Representative phase-contrast image of **e** GANT61-oligodendrocytes and **f** myelin basic protein (MBP) staining of GANT61-oligodendrocytes. **g** The representative phase-contrast image of co-culture GANT61-oligodendrocytes with rat DRG. The cell morphology of two GANT61-oligodendrocytes (yellow arrow) projected their processes toward the axon of rat DRG (red arrow). The asterisk indicates where the oligodendroglial membrane initiated to enwrap axonal process, which appeared as the thicker patterns comparing to thinner bare axon or non-myelinated segments. **h** Multilayered myelin is seen in co-cultures GANT61-oligodendrocytes with rat DRG under TEM (*n* = 3). **i** A higher magnification image of the myelin sheath showing light and dark bands. **j** The formation of compact myelin sheath in shiverer mouse brain after transplanted with GANT61-OPCs in the corpus callosum (*n* = 3). **k**, **l** Higher magnification images of the compact myelin sheaths. **m** Transplanted cells were detected in the shiverer mouse brain (in the corpus callosum) by co-labeling with anti-human nuclei antibody (red) and anti GFP stain (green) 2 weeks after transplantation. Error bars indicate SD, “A” indicates axon. Scale bar **g** 10 μm; **h** 500 nm; **i**, **k**, **l** 100 nm; **j** 1 μm

To understand the mechanistic insight into the pathways that may have contributed to the early maturation of GANT61-derived oligodendrocytes, we performed single-cell whole transcriptomic expression profiling on GANT61-OPCs (identified by PDGFR-α+, days 39–40). Two hundred thirty-four genes were identified as showing significantly different expression levels in response to GANT61 (FDR < 0.01). Of these, 24 genes were found to be upregulated (logFC > 1.2) and 49 genes as downregulated (logFC < 1.2) (Fig. 4a, Additional file 7). The GO terms that were found to be significantly enriched in the GANT61-OPCs were “cytoskeleton organization (GO:0007010),” “cellular response to transforming growth factor beta stimulus (GO:0071560),” “stress fiber (GO:0001725),” “filopodium (GO:0030175),” “ruffle membrane (GO:0032587),” “actin binding (GO:0003779),” and “laminin binding (GO:0043236)” (Fig. 4b, Additional file 8). On the other hand, the GO significantly downregulated in GANT61 were “basement membrane (GO:0005604)” and “intermediate filament cytoskeleton (GO:0045111)” (Fig. 4c, Additional file 9). Among the upregulated genes, particularly actin-related protein 2/3 complex subunit (also known as ARPC2 and ARP2/3), metallopeptidase inhibitor 1 (TPM1), integrin subunit beta 1 (ITGB1), and dystonin (*DST*) were of particular interest (see the expression of individual genes from single-cell transcriptomic analysis, Fig. 4d). The actin filament assembly drives the oligodendroglial process extension and ensheathment of the axon, they are important for the early stages of the oligodendroglial process branching [32–34]. The upregulation and interaction of laminin on β1 integrin promote oligodendroglial survival [35, 36] and formation of myelin sheath [37]. On the other hand, the reduction in *DST* expression found in GANT61-OPCs may associate with the halt of OPC, but not related to oligodendroglial maturation as previously described by Hossain et al. [38]. Thus, the effects of early transient inhibition of *GLI1* in NSCs on subsequent differentiation into oligodendrocytes seems to involve the greater activation of the pathways that promote cytoskeleton rearrangement geared toward initiation of the formation of mature branched structures and enhance motility.

### GANT61-NSC-derived oligodendrocytes are capable of forming multilayered myelin in vitro and in vivo

Following terminal differentiation, GANT61-oligodendrocytes differentiated into the cells with extensive sheet-like appearance and expressed myelin basic protein (MBP) (Fig. 4e, f). The myelination capacity of GANT61-oligodendrocytes was tested in the rat dorsal root ganglion cells (DRGs) co-culture model (*n* = 3), and also after transplantation into myelin-deficient shiverer mice’s brain (*n* = 3). In the co-culture model, the GANT61-derived late OPCs expressing the O4 marker (Fig. 4g) were plated on top of the rat DRG neurons and cultured in GDM medium for 3 weeks. Transmission electron microscopy (TEM) of the sections of the co-cultures (Fig. 4h, i) showed the presence of multilayered myelin around DRG axons. In the OLIG2-GFP knockin hESC line, GANT61-treated group generated functional OLs which were able to myelinate rat DRG neurons in the co-culture system (see Additional file 5: Figure S5.A).

We also checked the myelination capacity of GANT61-derived OL to form compact myelin in shiverer mice. Shiverer mice lack myelin basic protein (MBP) and therefore are incapable of making compact multilayered myelin. GANT61-derived O4^+^ OPCs transplanted into the corpus callosum of myelin-deficient mouse shiverer mice were capable of forming multilayered myelin structures when studied under an electron microscope 6 weeks after transplantation (Fig. 4j–l). The survival of the transplanted cells was tested in shiverer mice by the presence of anti-human nuclear stain and anti-GFP co-labeled cells in the corpus callosum 1 week after transplantation (Fig. 4m). GANT61-treated OLIG2-GFP H9 hESC-derived O4^+^ OPCs after transplantation into the corpus callosum of MBP-deficient shiverer mice were able to mature into MBP-producing oligodendrocytes after 4 weeks (Additional file 5: Figure S5. B). OPCs were detected in the corpus callosum by anti-GFP antibody (green cells), and they co-labeled with MBP stain (red).

## Discussion

In this study, we show that partial inhibition of GLI1 in hiPSC or hESC during neural induction give rise to NSCs that can differentiate earlier to mature functional oligodendrocytes in vitro. We had adopted a previously described protocol for the generation and isolation of OPCs from hiPSCs by tracking the expression of OLIG2 gene with the GFP reporter [12]. Adherent hiPSC colonies were concurrently exposed to RA and dual SMAD inhibitors, SB431542 and LDN193189, to generate PAX6-positive NSCs, which were then patterned toward OLIG2- and NKX2.2-expressing glial progenitors using RA and the Smoothened agonist SAG. These OLIG2-positive glial progenitors can be expanded as oligospheres and differentiated into pre-oligodendrocytes (identified by the expression of the O4 marker) and eventually became mature OLs (identified by expression of MBP) by day 50 and day 75, respectively. This protocol allowed us to explore the role of temporal partial inhibition of GLI1 signaling during NSC induction or during glial induction on the timing of OLIG2-positive OPC generation and maturation since the SHH signaling pathway has been shown to be involved in oligodendrocyte fate determination and maturation [3, 6, 19, 26–28].

First, we confirmed the expression of SHH pathway genes in human iPSCs (and hESCs) during the formation of NSCs and their subsequent lineage restriction into OLIG2-expressing glial progenitors. We confirmed the earlier findings that there is a significant upregulation of GLI1 during the generation of NSCs. We found that GANT61 decreased the mRNA levels of GLI1 significantly without changing GLI2 levels (similar to the results seen by Samanta et al. [23]).

Following initial partial inhibition of GLI1 with GANT61, the SHH pathway was later restimulated with the Smoothen agonist purmorphamine in order to induce OLIG2 expression. Both control and GANT61-NSCs gave rise to OLIG2^+^/GFP^+^ glial progenitors. When the GFP^+^ OPCs were analyzed by flow cytometry, a higher percentage of cells emerged in GANT61-OPCs which showed “high” GFP (OLIG2) expression. This was confirmed with RT-PCR analysis which showed that GANT61-derived cells had significantly higher mRNA levels of the key oligodendroglial fate-determining transcription factors genes OLIG2, as well as of NKX2.2 and SOX10. After differentiation, the GANT61-derived cells, however, generated similar numbers of PDGFR-α^+^ OPCs when compared to control-OPCs indicating that GANT61 did not modify the appearance of early OPCs. After further differentiation, however, GANT61-OPCs gave rise to a significantly greater number of pre-oligodendrocytes (~ 31.5% O4-expressing cells) compared to control OPCs which generated ~ 6.5% O4^+^ cells at day 45. After further differentiation, the fraction of the cells expressing mature OL markers and GALC^+^ was around three- and fivefolds higher in the GANT61-treated group.

By single-cell whole transcriptome expression profiling, we found that OPCs generated from NSCs in which GLI1 was transiently and partially inhibited had activation of genes in the pathways that promote cytoskeleton rearrangement geared toward the initiation of the formation of mature phenotypes (branched structures and enhance motility). Specifically, we performed the single-cell RNA sequencing assay (RNA-Seq) from PDGFR-α-expressing OPCs from the control and GANT61 groups. We also found that the genes associated with the cytoskeletal reorganization such as ARP2/3, TIMP1, and ITGB1 were activated in GANT61-OPCs compared to control-OPCs. This was particularly relevant since oligodendrocyte differentiation is a dynamic process involving high protrusion remodeling, with cells undergoing extreme cytoskeleton rearrangements that follow a cellular shaping program that governs morphological differentiation [32, 35, 36]. Typically, the oligodendrocytes undergo an initial period of protrusion remodeling shown by multiple retractions and extension of the filopodial, followed by the complex branched structure characteristic of pre-myelinating oligodendrocytes [1]. Actin polymerization is shown to be the key driver for the extension of membrane protrusions observed during terminal maturation of oligodendrocytes while the ARP2/3 complex, a key nucleator of actin networks found in lamellipodia, is required for the extension of oligodendrocyte protrusions [34, 39–41]. Previous reports have studied the role of the ARP2/3 complex in the migration of progenitor cells found in the brain. ARP2/3 is required for the directed migration of NSC-derived oligodendrocyte precursor cells (OPCs) in an electric field [40]. ARPC2-null OPCs were found to be slower and have shorter filopodial processes than ARPC2-expressing OPCs [40].

GANT61-derived oligodendrocytes were shown to be functional and were able to generate multilayered myelin sheath around the DRG neurons (in vitro) and in the corpus callosum of myelin-deficient shiverer mice (*n* = 3) after in vivo transplantation.

Recently, Samanta et al. [23] identified a subset of adult SHH-responsive pool of NSCs in the mouse SVZ that are recruited specifically to the demyelinated corpus callosum where they downregulate *GLI1* and differentiate into mature, myelinating oligodendrocytes. They compared the effects of partial and complete downregulation of *GLI1* expression by fate mapping NSCs in GLI1 null (GLI1CE/nLacZ; RCE) vs. GLI1 heterozygous (GLI1CE/+; RCE) mice. The major effects of partial loss of *GLI1* in NSCs appeared to be enhanced differentiation to oligodendrocytes. Also, partial inhibition of *GLI1* in NSCs (by GANT61-mediated pharmacological inhibition) promoted the maturation of these oligodendrocytes and recovery after demyelinating injury. Their observation that partial downregulation of *GLI1* expression was critical in NSCs to augment maturation and remyelination contrasted with the studies reporting that SHH signaling promoted remyelination after CNS injury and is required for remyelination. Interestingly, they found that complete inhibition of *GLI1* was ineffective in promoting remyelination, indicating that the role of GLI1 both in augmenting hedgehog signaling and in retarding myelination is dose-dependent and highly specialized. Their study and our current study indicate that the effects of loss of *GLI1* in NSCs appear to be enhanced differentiation to oligodendrocytes. Also, partial loss of *GLI1* has an even greater effect in the context of active SHH signaling, promoting robust recruitment and relieving an arrest of NSC differentiation into oligodendrocytes. This is in line with previous studies also showing that increasing the levels of SHH in the brain enhances the generation of OPCs but blocks their maturation.

## Conclusion

Our results with human iPSC show that partial inhibition of *GLI1* in hESC-derived NSCs followed by the activation SHH signaling in NSC differentiation toward OPCs leads to earlier maturation of oligodendrocytes in vitro. Our small molecule-based oligodendrocyte differentiation protocol is faster than all protocols reported so far. iPSC technology is fast developing as a tool for exploring new drug targets and gaining mechanistic insight into neurological disease. Development of iPSC-derived protocols that lead to efficient and faster differentiation of oligodendrocytes from neural progenitors provides important advances toward the development of autologous neural progenitor cell-based therapies

## Additional files

Additional file 1: **Figure S1.** (A) Cytotoxicity curve of GANT61 in iPSC (*n* = 3). Cells were treated with various doses of GANT61 for 5 days. Cytotoxicity was measured on day 6. (B) Dose-dependent reduction GLI1 mRNA in iPSC by a 5-day treatment with GANT61. (C) SHH pathway gene expression pattern of undifferentiated hESC, hESC derived-NSCs, and OLIG2-OPCs (*n* = 3, *t* test). (D) The quantification of nestin+, and (E) SOX2+ NSCs derived from OLIG2-GFP hESCs, show that similar number of NSCs were generated from both groups (control vs. GANT61). Immunofluorescence staining was done on day 5 of the differentiation timeline (*n* = 3). The percentages were calculated by counting the total number of DAPI-positive cells and the number of SOX2 or nestin-positive cells. Error bars indicate SEM. n.s = not significantly different. (TIF 844 kb)

Additional file 2: **Figure S2.** (A-E) Expression levels of genes in the SHH pathway in OLIG2/GFP-hiPSC derived OPCs upon the restimulation with SHH agonist purmorphamine (*n* = 3). (F, G) The representative flow cytometry data of the NG2+ population from GANT61-OPCs and control-OPC and the generation of OLIG2-GFP OPCs (*n* = 3). (TIF 171 kb)

Additional file 3: **Figure S3.** The cell viability of (A) OLIG2-NSCs and (B) OLIG2-OPCs. Only live-cells (propidium iodide negative, right panel) were selected and analyzed their dividing population based on the fluorescence dye dilution (*n* = 3 independent experiments). (TIF 465 kb)

Additional file 4: **Figure S4.** (A) Control- and GANT61-treated H9 OLIG2-GFP-hESC line-derived oligospheres expressing OLIG2 (green spheres). OLIG2-GFP-hESC were treated with GANT61 from day 0 to 5. Spheres were plated on day 24 and early OPCs migrated out within 48 h. Images are representations of 3 independent experiments. (TIF 6029 kb)

Additional file 5: **Figure S5.** (A) GANT61-treated hESC gave rise to functional oligodendrocytes. O4-positive OLs co-cultured with rat DRG neurons were able to myelinate them (*n* = 3, images have been taken from one experiment). MBP (red immune stain) co-labeled with SMI312 (magenta)-labeled neuronal tracts. The OLs were identified with anti-human nuclei antibody (green stain) which co-labeled with DAPI. (B) GANT61 treated hESC-derived OPCs were transplanted into the corpus callosum of the myelin deficient shiverer mouse. After 4 weeks, transplanted cells were identified with anti-GFP antibody (green) (*n* = 3). The MBP-positive cells (red) co-labeled with the GFP-expressing cells indicating that the OPCs matured into MBP producing oligodendrocytes. (TIF 2010 kb)

Additional file 6: **Table S1.** List of primers used in this study. (DOCX 15 kb)

Additional file 7: Upregulated and downregulated genes. (XLSX5 31 kb)

Additional file 8: GO terms that were found to be significantly enriched in the GANT61-OPCs. (XLSX 12 kb)

Additional file 9: GO significantly downregulated in GANT61-OPCs. (XLSX 11 kb)

## Data Availability

All data generated or analyzed during this study are included in this published article [and its supplementary information files].

## Abbreviations

Arp2/3: Actin-related proteins 2 and 3
DMSO: Dimethyl sulfoxide
DRG: Dorsal root ganglion
GALC: Galactosylceramidase
GFAP: Glial fibrillary acidic protein
GFP: Green fluorescent protein
GLI1: Glioma-associated oncogene homolog 1
GLI2: Glioma-associated oncogene homolog 2
GLI3: Glioma-associated oncogene homolog 3
hESC: Human embryonic stem cell
hPSC: Human pluripotent stem cell
iPSC: Induced pluripotent stem cell
MAG: Myelin-associated glycoprotein
MBP: Myelin basic protein
MOG: Myelin oligodendrocyte glycoprotein
mRNA: Messenger RNA
NSC: Neural stem cell
O4: Oligodendrocyte marker O4
OPC: Oligodendrocyte precursor cell
PDGFR-α: Platelet-derived growth factor receptor A
PLP: Myelin proteolipid protein
PTCH1: Patched 1
RNA-Seq: RNA sequencing
RT-PCR: Reverse transcription polymerase chain reaction
SHH: Sonic Hedgehog
SMO: Smoothened
SVZ: Subventricular zone
TF: Transcription factor
