# Supplementary figures and images for: Temporal and partial inhibition of GLI1 in neural stem cells (NSCs) results in the early maturation of NSC derived oligodendrocytes in vitro
Source: Stem Cell Res Ther. 2019 Aug 27;10:272. doi: 10.1186/s13287-019-1374-y (PMC6712625; doi:10.1186/s13287-019-1374-y)

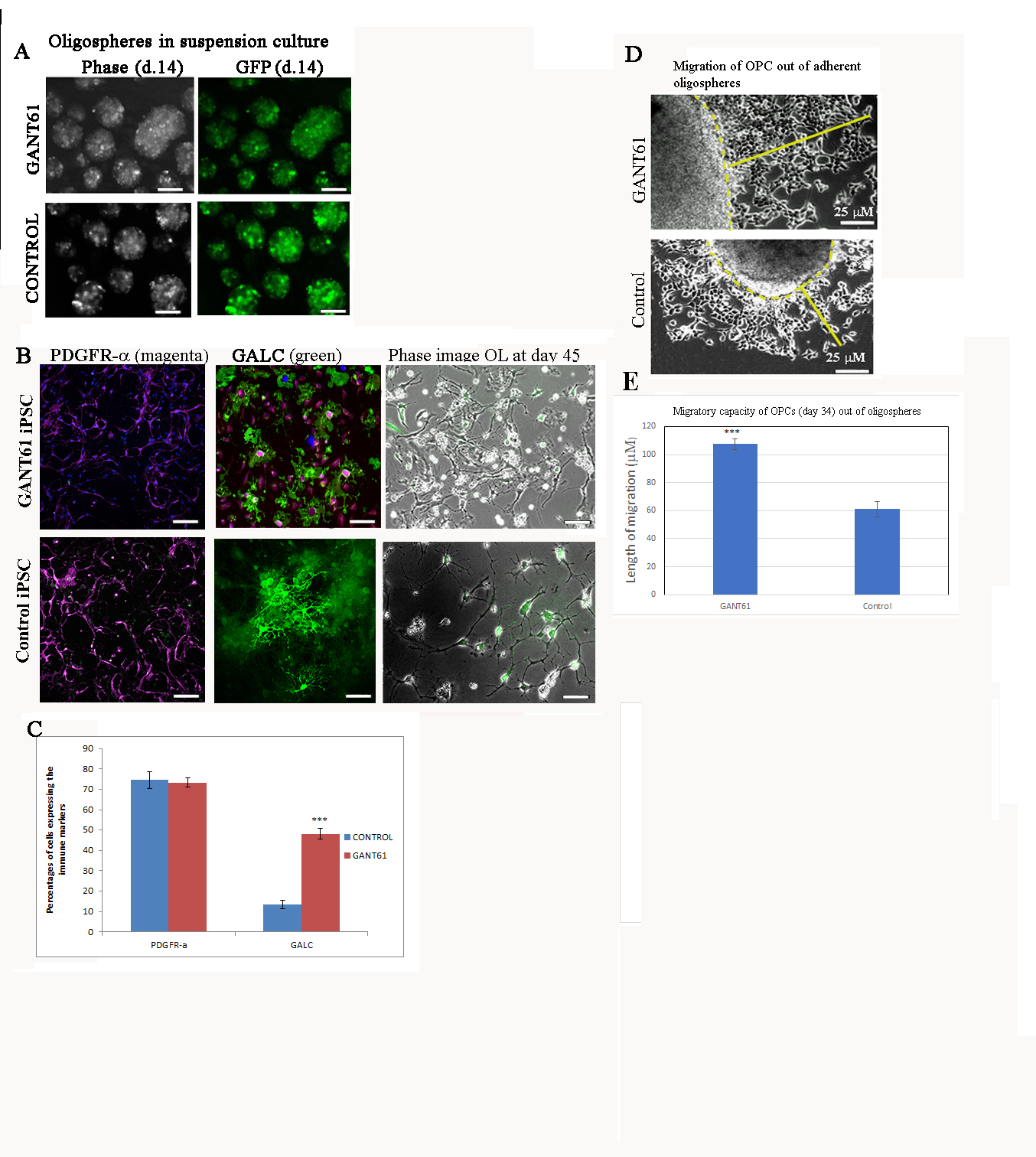

Supplement: Supplementary file 4 — Figure S4. (A) Control- and GANT61-treated H9 OLIG2-GFP-hESC line-derived oligospheres expressing OLIG2 (green spheres). OLIG2-GFP-hESC were treated with GANT61 from day 0 to 5. Spheres were plated on day 24 and early OPCs migrated out within 48 h. Images are representations of 3 independent experiments. (TIF 6029 kb) [file 13287_2019_1374_MOESM4_ESM.tif]

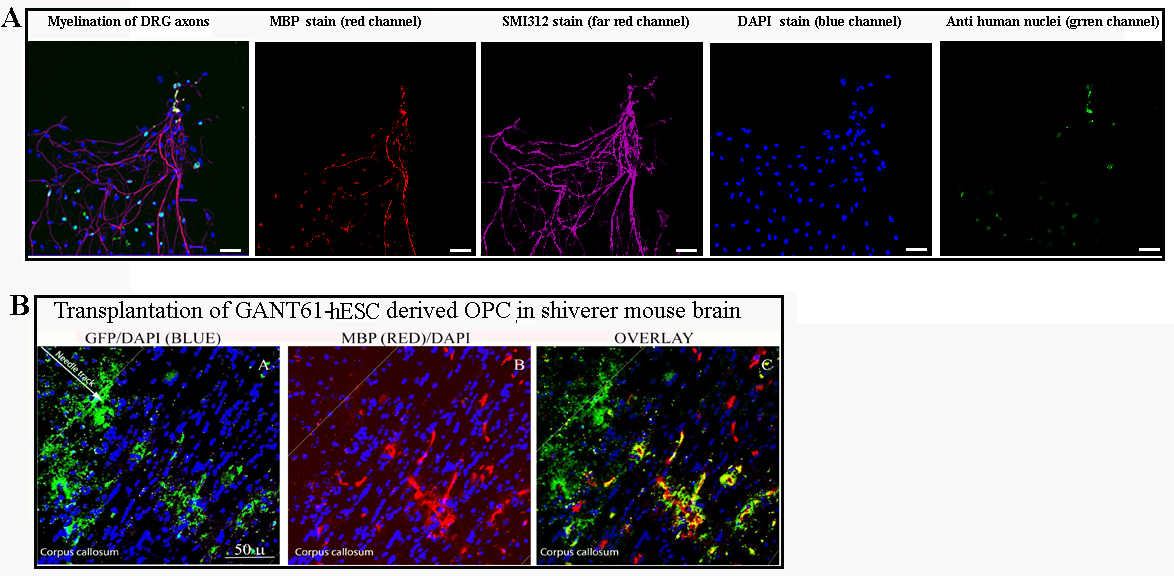

Supplement: Supplementary file 5 — Figure S5. (A) GANT61-treated hESC gave rise to functional oligodendrocytes. O4-positive OLs co-cultured with rat DRG neurons were able to myelinate them (n = 3, images have been taken from one experiment). MBP (red immune stain) co-labeled with SMI312 (magenta)-labeled neuronal tracts. The OLs were identified with anti-human nuclei antibody (green stain) which co-labeled with DAPI. (B) GANT61 treated hESC-derived OPCs were transplanted into the corpus callosum of the myelin deficient shiverer mouse. After 4 weeks, transplanted cells were identified with anti-GFP antibody (green) (n = 3). The MBP-positive cells (red) co-labeled with the GFP-expressing cells indicating that the OPCs matured into MBP producing oligodendrocytes. (TIF 2010 kb) [file 13287_2019_1374_MOESM5_ESM.tif]
