# Supplementary material for: Temporal and partial inhibition of GLI1 in neural stem cells (NSCs) results in the early maturation of NSC derived oligodendrocytes in vitro
Source: Stem Cell Res Ther. 2019 Aug 27;10:272. doi: 10.1186/s13287-019-1374-y (PMC6712625; doi:10.1186/s13287-019-1374-y)
Supplement: Supplementary file 6 — Table S1. List of primers used in this study. (DOCX 15 kb) [file 13287_2019_1374_MOESM6_ESM.docx]

**Table1:** List of primers used in this study

| Target gene | Forward primer 5’>3’ | Reward primer 5’>3’ |
| --- | --- | --- |
| GAPDH | ACCACAGTCCATGCCATCAC | GTCAGGTCCACCACTGACAC |
| GLI1 | AGCGTGAGCCTGAATCTGTG | CAGCATGTACTGGGCTTTGAA |
| GLI2 | CTGCCTCCGAGAAGCAAGAAG | GCATGGAATGGTGGCAAGAG |
| GLI3 | GAAGTGCTCCACTCGAACAGA | GTGGCTGCATAGTGATTGCG |
| OLIG2 | CCAGAGCCCGATGACCTTTT | TCCGGCTCTGTCATTTGCTT |
| MBP | CCGGCAAGAACTGCTCACTA | CGTCTAGCCATGGGTGATCC |
| MCSP4 | CACGGCTCTGACCGACATAG | CCCAGCCCTCTACGACAGT |
| NESTIN | GAAGGGCAATCACAACAGGTG | GGGGCCACATCATCTTCCA |
| PATCH1 | GAAGAAGGTGCTAATGTCCTGAC | GTCCCAGACTGTAATTTCGCC |
| PLP1 | ACCTATGCCCTGACCGTTG | TGCTGGGGAAGGCAATAGACT |
| PDGFαR | TTGAAGGCAGGCACATTTACA | GCGACAAGGTATAATGGCAGAAT |
| SMO | TCGAATCGCTACCCTGCTG | CAAGCCTCATGGTGCCATCT |
| SOX10 | CCTCACAGATCGCCTACACC | CATATAGGAGAAGGCCGAGTAGA |
